# Letter to: Real‐World Clinical Experience With Serum MOG and AQP4 Antibody Testing by Live Versus Fixed Cell‐Based Assay

**DOI:** 10.1002/acn3.70062

**Published:** 2025-05-14

**Authors:** Adrian Budhram

**Affiliations:** ^1^ Department of Clinical Neurological Sciences Western University, London Health Sciences Centre London Ontario Canada; ^2^ Department of Pathology and Laboratory Medicine Western University, London Health Sciences Centre London Ontario Canada

I read with interest this manuscript by Said et al. [1] which reports the sensitivity of anti‐MOG and anti‐AQP4 fixed CBA to be substantially lower than that of live CBA (approximately 50% lower for anti‐MOG and 25% lower for anti‐AQP4). This contrasts with several prior reports, which describe a more modest 10%–15% lower sensitivity of anti‐MOG fixed CBA and comparable sensitivity of anti‐AQP4 fixed CBA when compared to live CBA [2, 3]. The authors appropriately acknowledge these discrepancies and note that it is conceivable that differences in laboratory practices and training may be a contributor, even though fixed CBA was performed at a large academic center. Could the authors elaborate on fixed CBA testing at this center, including what instrumentation is used to run samples, whether manual or automated microscopy is used to read slides, the number of readers employed, and whether any grading of immunofluorescence (e.g., Weak Positive, Positive, 1+, 2+, etc.) is reported?

The authors also state that testing by both fixed and live CBA was not performed for all patients with suspected demyelinating attacks, but that they would not expect this to significantly impact estimates of specificity/sensitivity because these measures are not dependent on disease prevalence in the tested population. However, estimates of specificity/sensitivity are susceptible to bias arising from suboptimal selection of the tested population [4]. The authors state that one typical scenario for testing samples by both fixed and live CBA was a persistently high index of suspicion despite negative fixed CBA testing locally. If the proportion of patients who underwent testing by both assays for this reason was high, then this would intuitively seem to be biased against the calculated sensitivity of fixed CBA relative to live CBA; it could enrich your tested population with patients who are negative by fixed CBA but positive by live CBA, and deplete your tested population of patients who are positive by fixed CBA but negative by live CBA. This potential bias may contribute to the significant discrepancy in the proportion of samples that were positive for anti‐MOG by fixed CBA but negative by live CBA in their clinical testing cohort versus biobank cohort (1/552 [0.2%] versus 4/42 [9.5%], *p* = 0.0001 by Fisher's exact test). Could the authors elaborate on the indications for testing by both fixed and live CBA in their cohort, and in particular clarify what proportion of patients tested by both assays were initially negative by fixed CBA?

## Author Contributions

A.B. contributed to drafting the manuscript.

## Conflicts of Interest

Adrian Budhram reports that he holds the London Health Sciences Centre and London Health Sciences Foundation Chair in Neural Antibody Testing for Neuro‐Inflammatory Diseases. He receives support from the Opportunities Fund of the Academic Health Sciences Centre Alternative Funding Plan of the Academic Medical Organization of Southwestern Ontario (AMOSO).

## Data Availability

Data sharing is not applicable to this article as no new data were created or analyzed in this study.
